# The development of positive education combined with online learning: Based on theories and practices

**DOI:** 10.3389/fpsyg.2022.952784

**Published:** 2022-08-30

**Authors:** Jialing Lou, Qinmei Xu

**Affiliations:** ^1^Learning and Cognitive Science Laboratory, Interdisciplinary Center for Learning and Cognitive Science, College of Education, Zhejiang University, Hangzhou, China; ^2^The Children’s Hospital, and National Clinical Research Center for Child Health, Zhejiang University School of Medicine, Zhejiang University, Hangzhou, China; ^3^Zhejiang University-Jiaxing Joint Center for Mental Health, College of Education, Zhejiang University, Hangzhou, China

**Keywords:** positive education, positive psychology, online learning, the PERMA model, wellbeing

## Abstract

In recent years, increasing attention has been paid to the all-around development and mental health of students in education. Positive education, a rapidly developing ramification of positive psychology, has proved beneficial to students’ learning and wellbeing. Meanwhile, online learning has quickly gained popularity due to the impact of COVID-19. However, there have been few reports discussing the relationship between positive education and online learning by combining theories and practices. To explore the connection between positive education and online learning, we provide a literature review for studies, mostly between 2010 and 2022, of theories and practices for both positive education and online learning. Next, we establish one-to-one links between the relevant theories and practices of online learning to each domain in the PERMA model of positive education, a theoretical framework including Positive emotion(P), Engagement(E), Relationship(R), Meaning(M), and Accomplishment(A). We aim to explore how to promote the development of positive education by applying the theoretical and practical advantages of online learning to the PERMA framework of positive education. This study aims to enrich the research perspectives of positive education and provide a reference for future research.

## Introduction

With the rising risk of mental health disorders among young people, school education is increasingly focusing on students’ mental health (Noam and Hermann, 2002; Weist and Murray, 2008; Atkins et al., 2010). All of these have prompted placing wellbeing and academic development as equally valued core priorities in education (Bonell et al., 2014; Suldo et al., 2014; Kern and Wehmeyer, 2021). Positive education evolving fast in the last decade, which encompasses multiple theories, programs, frameworks, and approaches (Slemp et al., 2017), can develop both traditional academic skills and happiness (Seligman et al., 2009). The PERMA wellbeing model, proposed in positive psychology, includes Positive emotion(P), Engagement(E), Relationship(R), Meaning(M), and Accomplishment(A), has been applied to the practice of positive education (Hoare et al., 2017; Seligman, 2018). This model is one of the important basic theories of positive education (Kern et al., 2015, 2016; Noble and McGrath, 2015; Norrish, 2015; White and Murray, 2015; Waters and Loton, 2019).

The constant update of information technology promotes the rapid development of online learning (Danchikov et al., 2021). Online learning is a form of non-face-to-face education that relies on computers, the Internet, and other relevant information communication technologies (Salmon, 2011). Some scholars have pointed out that online learning has rich theoretical foundations, including behaviorism, cognitivism, constructivism, connectionism, and heutagogy (Johnson and Aragon, 2003; Ally, 2008). Different learning and teaching theories all play key roles in the design and implementation of online learning; for example, cognitivism can be used to guide the design of online teaching materials and constructivism can be used to guide the design of situated learning environments (Blaschke, 2012; Goldie, 2016). The combination of various theories can take advantage of their strong points and develop more effective online learning. At the same time, online learning is becoming a terrific complement to the original offline education under the impact of COVID-19 (Dhawan, 2020; Sutiah et al., 2020). Currently, online learning can be divided into synchronous online learning, asynchronous online learning, or hybrid online learning according to whether the interaction time is synchronous (Hrastinski, 2008). It can also be classified into interactive online learning (Elsamanoudy et al., 2020), collaborative online learning (Kumi-Yeboah, 2018), and adaptive online learning (Komleva and Vilyavin, 2020) by the form of interaction.

In recent years, some researchers pointed out that one of the future development trends of positive education is to combine with technologies such as the Internet (Burns and Weinberg, 2017). Some attempts have been made to promote the development of positive education. For example, “HQ thrive,”^1^ an online positive education website in Australia, combines the advantages of online learning, and creates a platform for educators and learners to learn independently. Meanwhile, some scholars have proposed an online positive education program that provides an online community where students can interact with each other (Morgan and Simmons, 2021). It is becoming an irreversible trend to carry out positive education online, which will further promote the science and sustainability of positive education.

As online learning promotes the development of positive education, there have been few reports discussing the relationship between positive education and online learning based on theories and practices. To explore the connection between positive education and online learning, this paper will first review the research on positive education and online learning. Then, we elaborate how to establish one-to-one links between the domains of the PERMA model of positive education, and theories of learning related to online learning (behaviorism, cognitivism, constructivism, connectionism, and heutagogy). At the same time, to enhance the practicality of the ideas, some existing practices and techniques of online learning will be used to illustrate how to promote practices of positive education.

## Theoretical model and practice of positive education

### What is positive education

Positive education originated from positive psychology. Positive psychology is the science of positive subjective experience (e.g., happiness), positive individual traits (e.g., talents, interests, and strengths of character), positive relationships (e.g., friendship), and positive institutions (e.g., families, schools, and communities; Seligman, 2002). Positive psychology works to find effective mechanisms that can help individuals, groups, and organizations to promote wellbeing through scientific research methods, thereby promoting people to achieve a flourishing state (Seligman and Csikszentmihalyi, 2014). Positive education is defined as the education for both traditional skills and happiness (Seligman et al., 2009). There are a variety of definitions of positive education in academia. Oades et al. (2011) describe positive education as the application of positive psychology in education. Norrish et al. (2013) consider that positive education brings together the science of positive psychology with best-practice teaching to encourage and support schools and individuals within their communities to flourish. Slemp et al. (2017) state that positive education aims to build strengths, capabilities, wellbeing, and resilience in educational communities. Although these definitions differ in presentation, they all emphasize positive education as an applied science that weaves positive psychology into educational practice intending to promote wellbeing and other positive states and qualities (Waters and Loton, 2019).

### The theoretical model of positive education

Positive education encompasses multiple theories and frameworks, and there is no single theoretical model that fully represents the field. A typical wellbeing theory in macro positive psychology is the PERMA model. The PERMA model shows five pillars of wellbeing: Positive emotion, Engagement, Relationships, Meaning, and Accomplishment (Seligman, 2011). Since then, Geelong Grammar School has further enriched the PERMA model by including physical health (H) and therefore the PERMA-H model. The model provides a structured pathway for implementing positive education, a framework to guide evaluation and research, and a foundation for further theoretical development (Norrish et al., 2013). Noble and McGrath (2015) further proposed the PROSPER framework. The framework highlights seven key elements that contribute to wellbeing: Positivity, Relationships, Outcomes, Strengths, Purpose, Engagement, and Resilience. Some scholars also suggested a new model named EPOCH based on the PERMA model. The EPOCH framework consists of five elements: Engagement (E), Perseverance (P), Optimism (O), Connectedness (C), and Happiness(H) (Kern et al., 2016). In addition, many other new theoretical frameworks are emerging in the field of positive education, such as the SEARCH framework [Strengths (S), Emotional management (E), Attention and Awareness (A), Relationships (R), Coping (C), and Habits and Goals (H) (Waters and Loton, 2019)].

Previous research has indicated that all five elements of the PERMA model can protect against negative emotions and physical illness (Kern et al., 2015), and also enhance resilience and life satisfaction in youths (Falecki et al., 2018). It can be considered that the PERMA model is the best approximation of what humans pursue for their own sake, which is why it has a place in wellbeing theories (Leontopoulou, 2020). Kern et al. (2015) pointed out that the PERMA model could suitably assess dimensions that are valued by youth while also aligning to existing school structures and strategies. It is arguably the most popular framework in education (Waters and Loton, 2019). On the one hand, the PERMA model has been widely used in education with a study population spanning different age groups (Oades et al., 2011; Buchanan et al., 2022). On the other hand, it has been practiced in different countries and regions around the world, such as Australia, Indonesia, China, Turkey, and so on (Ayse, 2018; Hidayat, 2018; Gray et al., 2020; Cheng and Chen, 2021). In particular, it has been used as overarching positive education framework by some notable positive education schools, such as St Peter’s College, Adelaide (White and Murray, 2015), and Geelong Grammar School (Norrish, 2015). In addition, PERMA model as a classic theory proposed earlier in the field of positive education has been developed on the basis of PERMA, such as EPOCH Model (Kern et al., 2016) and PROSPER model (Noble and McGrath, 2015).

However, the PERMA model still needs more measuring objective indices, longitudinally as well as synchronously, and testing the influence of interventions in the future (Seligman, 2018). In short, PERMA was hailed as a framework, particularly suited to examine multiple dimensions and patterns of wellbeing in positive education (Leontopoulou, 2020).

### The practice of positive education

Over the past decade, the positive education movement has spread around the world (Seligman, 2019). Various organizations have emerged, such as the International Positive Education Schools Association,^2^ the International Positive Education Network,^3^ and so on. In addition, there are also notable projects and practices, such as the FRIENDS Program in the United Kingdom (Stallard et al., 2014), and so on.

There are numerous practices in positive education, which are diverse in theme and form (Morrish et al., 2018; Schiavon et al., 2020). White and Murray (2015) summarized three main forms of positive education: (1) Wellbeing intervention programs that are evidence-based and professionally supported; (2) Scientifically informed proactive strategies for the whole school mental health programs; and (3) Specific virtues or values and character-based school lessons based on philosophy or values-based learning. At the same time, the topics concerned with positive education practice can be categorized into levels of cognitive (e.g., values and mindset), emotion (e.g., happiness and optimism), and competence (e.g., resilience and wellbeing literacy). At the cognitive level, the “Believing You Can” program in Australia combines cognitive behavior therapy techniques to encourage students to critique their thought patterns, and to promote positive learning experiences for students (Chodkiewicz and Boyle, 2017). At the emotion level, studies proved that positive education intervention can reduce students’ negative emotions and the occurrence of depression (Waters, 2011; Zhao et al., 2019). At the competence level, Oades (2017) pointed out that positive education can cultivate students’ wellbeing literacy, the ability to have lasting wellbeing.

Positive education may have the following trends in the future, combined with a third wave of positive psychology (Lomas et al., 2021). With the expansion of epistemologies, more holistic, complex dynamic-systems approaches are being used to develop and implement positive education (Lomas et al., 2015). These approaches include: (1) a better understanding of context (e.g., historic, social, cultural, and institutional) (White and Murray, 2015; Ciarrochi et al., 2016; Joshanloo et al., 2021); (2) expansion to diverse populations, especially the inclusion of minority voices (Slemp et al., 2017; White, 2017); and (3) systems-informed perspectives, specifically referring to incorporating principles and approaches from the systems sciences into positive education. Systems-informed positive education embeds individuals within broader and more complex social systems including students, teachers, staff members, school leaders, parents, and others, to bring out the best in each and of the school community as a whole (Kern and Taylor, 2021). Meanwhile, there is a broadening of methodologies which includes qualitative and mixed methods (White and Kern, 2018), computational social science (Burns and Weinberg, 2017), and so on.

## Online learning

Online learning was first put forward in 1995, which is the process of teaching and learning in the Internet environment (Singh and Thurman, 2019). Compared to traditional offline education, online learning has numerous advantages, such as ease of teaching online, flexibility of work schedule, and few space limitations (Watson, 2008; Al-Zaidiyeen et al., 2010; Karasneh et al., 2021); adaptability to broad learning styles (Mehlenbacher et al., 2000; Daughenbaugh et al., 2002; Stern, 2004); variety of multimedia resources accessible (Bączek et al., 2021; Pelletier et al., 2021); ease in monitoring and documenting teaching activities (Gherheș et al., 2021); reduction in educational expenses (Nguyen, 2015; Batdi et al., 2021), and so on. However, online education also has some drawbacks, such as a lack of efficiency in receiving students’ responses to requests, procrastination, poor attendance, a sense of isolation, and less satisfied with the learning experience (Carr, 2000; Rivera et al., 2002; Wang et al., 2017; Heuberger and Clark, 2019; Danchikov et al., 2021). In 2019, the outbreak of COVID-19 has pushed the enthusiasm and application for online learning to a new peak (Pelletier et al., 2021). A World Bank report provides a general overview of the emergency responses of over 120 countries from April to May 2020 during the COVID-19 pandemic. The report shows most countries designed and implemented nationwide remote learning initiatives involving online programs (Barron Rodriguez et al., 2021). Another report from the European Commission states that, following COVID-19, the majority of teachers (66.9%) had to teach online for the first time (Di Pietro et al., 2020). Online learning has proved to be not only an important alternative to traditional education but also a key point for future education reform.

Online learning has a rich educational theoretical foundation, which involves many schools of thought, such as behaviorism, cognitivism, constructivism, connectionism, and heutagogy (Johnson and Aragon, 2003; Anderson, 2008). Adopting a synthesized theory of learning can have a synergistic result by integrating the advantage of each individual learning theory into online learning (Johnson and Aragon, 2003). The behaviorist school of learning, represented by Pavlov, Thorndike, and Skinner, believes that learning is an observable and measurable explicit behavioral change caused by external environmental stimuli (Skinner, 1974). In the early days of computers in education, programmed instruction as developed by behaviorist Skinner was usually applied to the design of educational software (Skinner, 1958; Smith-Gratto, 2000). A typical example is the design of computer-assisted instructions in early online learning which involves processes of identifying the goal, breaking that goal down into sequential learning steps, and providing feedback (or reinforcement) to students (Smith and Boyce, 1984). The utility and ease of programmed instruction derived from the behaviorist paradigm to keep students focused and headed toward certain educational objectives during online education cannot be understated (Smith-Gratto, 2000; Root and Rehfeldt, 2021). Compared with behaviorism, cognitivism emphasizes the inner formation of knowledge in students’ minds (Clark, 2018). Guided by cognitivism, the presentation of online learning materials focuses on linking to prior knowledge (Brieger et al., 2020) and preventing cognitive overload (van Merriënboer and Ayres, 2005). Constructivism believes that learning is the process of learners’ independent exploration and construction in the context with the help of collaboration and communication (Fosnot, 1996). Situated learning, a collaborative learning theory based on social constructivism, has been widely used in online learning (Woo and Reeves, 2007). Specifically, online learning builds learning pages in line with teaching themes based on situated learning, and uses virtual reality and other technologies to create virtual learning environments (Dawley and Dede, 2014). At the same time, online learning provides an ideal social space for collaborative learning based on the Internet platform, where students can form a knowledge community through partner cooperation and role-playing (Stacey, 1999). Connectivism is a conceptual framework which views learning as a network phenomenon influenced by technology and socialization (Siemens, 2004; Downes, 2005). According to the enlightenment of connectionism, online learning can entrust part of the learning task of memory and storage to intelligent agents, so that students can participate in knowledge sharing and communication more (Kop and Hill, 2008). Online learning not only helps students to connect to various network databases but also tries to connect learners with each other (Kropf, 2013). Therefore, various forms of interaction are increasingly used in the teaching design of online courses, such as online discussion, bullet screens, and so on (Reese, 2015). Online learning also involves the theory of heutagogy. Heutagogy emphasizes that learners, as study agents, decide learning objectives and processes according to their own needs in an autonomous learning environment that enriches the self-determination theory (Hase and Kenyon, 2000, 2007). The principles of heutagogy include learner agency, self-efficacy, and capability, reflection and meta-cognition, and nonlinear learning (Blaschke and Hase, 2019). Under the guidance of heutagogy, online learning takes more consideration of learners’ own needs and learning styles (Blaschke and Hase, 2016). Online learning can provide learners with a more flexible and autonomous learning environment which can stimulate students’ motivation and increase students’ stickiness of learning (Eberle and Childress, 2009).

## Online learning promotes positive education: Based on relevant theories and practices

**Figure 1:**
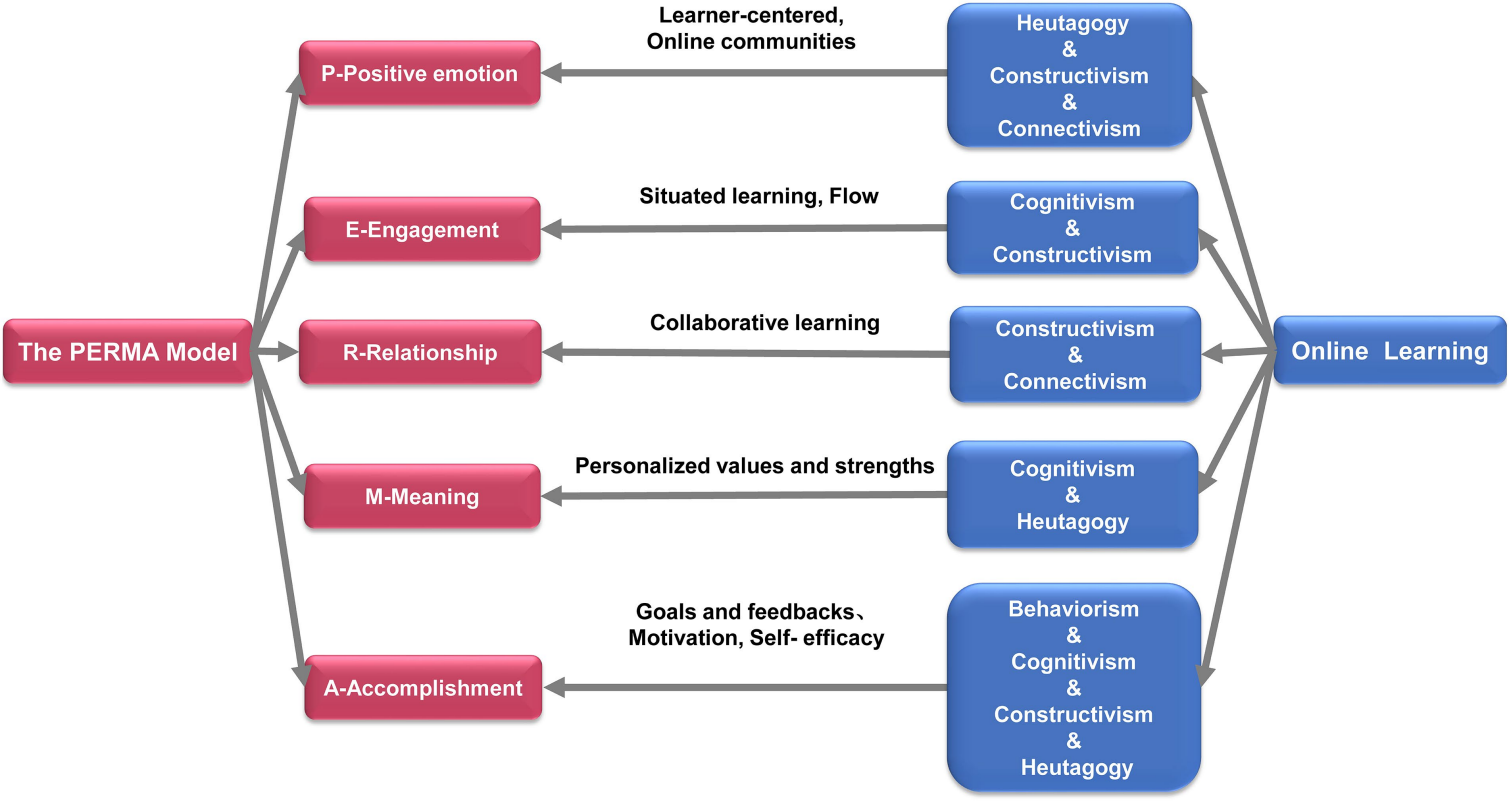
How does online learning promote the development of positive education.

Online learning, characterized by convenient dissemination and timely updating, has the potential to ensure the normal continuation of high-quality positive education under COVID-19 and other special situations (Adedoyin and Soykan, 2020). Carrying out positive education through online learning is one of the future trends of positive education. However, few reports have combined relevant theories and practices to elaborate the relationship between positive education and online learning (Morgan and Simmons, 2021; Khan and Thomas, 2022). In the following paragraphs, we will connect each component of the PERMA model to the theory and practice of online learning to elaborate how online learning promotes positive education in the future (Figure 1).

The first component of PERMA, the domain of “Positive emotion” focuses on students’ positive emotions, such as feelings of happiness, and motivation during learning (Seligman, 2011; Kern et al., 2015). Positive emotions play an important role in online learning. Some scholars define emotional presence as an experience that is salient in the online environment (Cleveland-Innes and Campbell, 2012). Zembylas (2008) states that positive emotions include joy, enthusiasm, and excitement for the flexibility of online learning; pride and contentment for fulfilling the course requirements; and surprise and excitement for the emotional nature of online communication. Heutagogy, as a learner-centered theory, emphasizes the development of learner autonomy and capacity during online learning (Blaschke, 2012). A recent study used constructivist digital learning heutagogy to reveal the relationship between positive education and online learning. The findings found that constructivist digital learning heutagogy can promote positive emotions (Khan and Thomas, 2022). It is also worth noting that emotion is often present in online learning communities (Derks et al., 2008; Marchand and Gutierrez, 2012). Therefore, constructivist and connectionist perspectives in online learning can be utilized to build more harmonious online learning communities to facilitate students’ online communication and emotional expression (Morgan and Simmons, 2021). MacFadden et al. (2005) proposed a constructivist model to online learning that emphasizes the emotional dimensions in instruction and on utilizing these dimensions in learning. There are also studies that address the use of online communities to meet students’ emotional needs from a constructivist view (Torres and Evans, 2020).

The second component of PERMA, the domain of “Engagement,” emphasizes feeling engaged in learning activities. The process of concentration produces flow, which is a highly focused state of total devotion (Csikszentmihalyi, 1990). Some studies have proved that game activities are easy to produce flow experience (Hamari et al., 2016). Constructivism in online learning emphasizes the importance of situated learning (Terhart, 2003). So that online positive education can make use of online learning to create a gamified positive education situation suitable for students’ learning, providing students with immersive learning experiences and improving their participation (Levine, 2007). For instance, there is already a positive intervention game for young people called REThink. REThink has been shown to foster mental resilience in children and adolescents aged 10 to 16, helping them learn wellbeing strategies to cope with abnormal negative emotions such as anxiety, anger, and depression (David et al., 2018, 2021). Another widely applied positive education program involving an online format is called “Bounce back!.” “Bounce back” is a series of interactive books, supported by online materials and games, which covers a range of curriculum units: Values, Resilience, Courage, Emotions, Relationships Bullying, and Humor (McGrath and Noble, 2003). There is also a recent study that specifically explores the relationship between positive education and online learning, and results reveals that learning through constructivist digital learning heutagogy supports positive emotions, academic achievement, and learning engagement (Khan and Thomas, 2022). In addition to games, developing self-learning materials and creating situated learning guided by constructivism also integrate the whole environment into students’ learning, and successfully provide multiple avenues for learning processes and eventually promote learner engagement (Wanniarachchi, 2016). It has also been found that multimedia online learning under cognitivism reduces students’ cognitive load and increases students’ learning engagement by elaborating video learning content that matches students’ cognition (Singh, 2022).

The third component of PERMA, the domain of “Relationship” refers to feeling socially supported, loved, and valued by others (Seligman, 2011). As an important theoretical basis of online learning, connectionism and social constructivism both emphasize the importance of building positive interpersonal relationships (Lain, 2016). Positive education combined with online learning can create learning communities through social media or meta-universe and build good communication and learning relationships among students. Some research evidence suggested the role of social networks in supporting collaborative e-learning based on connectivism theory has a positive impact on facilitating student–student and student-teacher interactions (Mallon, 2013; Thota, 2015; Alzain, 2019). Ansari and Khan (2020) revealed that online social media for collaborative learning can promote peer and teacher interaction. In response to concerns around mental health of students during the pandemic, some scholars designed and implemented an 8-week online wellbeing program based on positive education frameworks (the “PERMA” wellbeing model). The program provides students with an online community, an opportunity to feel connected with other students, and resources to improve wellbeing (Morgan and Simmons, 2021). It has also been proposed that the metaverse can increase the opportunities for students to communicate in learning and life by building a virtual community (Jovanović and Milosavljević, 2022).

The fourth component of PERMA, the domain of “Meaning,” refers to a feeling of belonging and/or serving something greater than ourselves and connecting with strengths and values can make learning more meaningful (Oades et al., 2011). Heutagogy in online learning can enhance the meaning of learning. One of the basic principles of heutagogy is learner agency, which empowers the student to take control of their learning by choosing what and how they learn based on their values (Blaschke, 2012, 2018). Nonlinear learning as another basic premise of heutagogy encourages learners to actively personalize their learning paths according to their own goals and capabilities (Peters, 2003; Blaschke, 2021). Cognitivism in online learning also highlights that a meaningful online learning must be relevant to the individual, such as learner’s prior knowledge, interests, and other relevant characteristics (Davidson-Shivers et al., 2018). Online learning can give play to technological advantages like artificial intelligence, learning analysis, intelligent recommendation and, big data to collect and analyze learner’s advantages, learning styles, and psychological status. It can explore the individual advantages for different students, so as to maximize the abilities of students (Xue et al., 2020), and stimulate the enthusiasm of students to actively participate in meaningful learning.

The fifth component of PERMA, the domain of “Accomplishment,” is a feeling of working toward and reaching goals, having motivation and self-efficacy to finish what one set out to do (Seligman, 2011). Fulfillment comes from working toward a goal and eventually achieving it. In the context of education, accomplishment means implementing assessments for learning and of learning (Oades et al., 2011). Behavioral learning theory often lend itself to instructional design based on very specific and discrete learning steps (Harasim, 2011). Programmed instructions guided by behaviorism take some online learning content with clear learning goals and cut it into small chunks of learning objectives, giving timely feedback based on student learning (Smith-Gratto, 2000). When combined with cognitivism, online learning can present learning materials and practices that are in line with zone of proximal development according to learner’s learning progress and cognitive level (Maravanyika et al., 2017). In constructivist contexts, teachers can provide more interactive feedback, such as scaffolding (Davidson-Shivers et al., 2018). In the meantime, student’s wellbeing can be quantified as a measurable goal during online learning. One study uses online positive psychology interventions to measure intentional emotional vocabulary use, quantifying wellbeing as explicit wellbeing literacy goals (Francis et al., 2020). Furthermore, adaptive learning can be adopted as a way of learning. According to the current knowledge level of students, when learning challenges with appropriate learning pace and difficulty are provided, they can fully mobilize the enthusiasm of students to learn and challenge themselves (Tseng et al., 2008). However, behavioral and cognitivism typically direct students to focus on learning and may ignore students’ own interests and motivation (Smith-Gratto, 2000). Heutagogy in online learning emphasizes learner agency, self-efficacy, and capability (Blaschke and Hase, 2019). Under the guidance of heutagogy, students can be intrinsically motivated to learn and to discover how they can best meet their learning objectives (Canning, 2010; Blaschke and Hase, 2015; Hartnett, 2016).

## Discussion

The growing focus on the all-round development of young people has led to the recognition of the significance of wellbeing and mental health (Chodkiewicz and Boyle, 2017). Positive education combines the principles of positive psychology with best practices of teaching and educational paradigms to promote the optimal development and prosperity of students. This paper expounds on the research and practice related to positive education, reviews theories of online learning. Then we explore how to promote the development of positive education by applying the theoretical and practical advantages of online learning to the PERMA framework of positive education.

Although the PERMA is a prolific model in positive education, there are still room for future improvements. The five elements of the PERMA model are exclusive, but certainly not exhaustive. Additional elements, such as health, vitality, and responsibility, are additional key candidate elements to be assessed in future (Goodman et al., 2018). Currently, little is known about which interventions have the most impact on the elements of PERMA. The effectiveness of interventions that primarily target a single element can suggest the relative importance of the newly proposed element (McQuaid and Kern, 2017). For example, does it make sense to build engagement without much impact? In addition, this article covers only five theories related to online learning. Other models and approaches could offer different additional possibilities. There are existing studies that combine the PERMA model with the Constructivist Digital Learning Heutagogy model (A model guided by online learning theory) to design positive educational interventions in the online format (Khan and Thomas, 2022). In the future, the possibility of correlating other theoretical models of online learning with the PERMA model of positive education could be further explored.

During post-epidemic era, online formats will continue to exist but no longer as the only form of emergency education, and hybrid education combining online and traditional formats may be further studied in the future (Lockee, 2021; UNESCO et al., 2021). Today’s adolescents are “digital natives,” who have been surrounded by digital technology since birth (Prensky, 2001). In other words, it is a direction of interest to improve online learning to suit the development of children and youth in the future. Still, studies showed that there exist more negative emotions among children in online learning due to the impact of the pandemic on children. One possible future study could focus on the exploration of children’s mental health in online learning, such as integrating more emotional factors (Zembylas, 2008). Additionally, improving the quality of online learning is also a topic worth exploring. There are many studies pointing out high dropout rates (Boston et al., 2012) and low academic completion (Teclehaimanot et al., 2007) during online learning. It is possible to consider incorporating flow theory to enhance student engagement in the online learning process.

In summary, combining the learning theories related to online learning can contribute to the development of PERMA model in positive education. But there are still some challenges about PERMA model and online learning. Researchers can combine more theoretical models to explore how to promote the development of different dimensions of the PERMA model, or add positive elements in online education to increase the sustainability of online education development.

## Author contributions

JL contributed to the conception and design of the article and wrote the first draft of the manuscript. QX contributed to manuscript revision, read, and approved the submitted version.

## Conflict of interest

The authors declare that the research was conducted in the absence of any commercial or financial relationships that could be construed as a potential conflict of interest.

## Publisher’s note

All claims expressed in this article are solely those of the authors and do not necessarily represent those of their affiliated organizations, or those of the publisher, the editors and the reviewers. Any product that may be evaluated in this article, or claim that may be made by its manufacturer, is not guaranteed or endorsed by the publisher.

## Footnotes

^1^ https://www.hqthrive.com

^2^ www.pesa.edu.au

^3^ www.ipen-network.com
